# TOSCA – first international registry to address knowledge gaps in the natural history and management of tuberous sclerosis complex

**DOI:** 10.1186/s13023-014-0182-9

**Published:** 2014-11-26

**Authors:** John C Kingswood, Paolo Bruzzi, Paolo Curatolo, Petrus J de Vries, Carla Fladrowski, Christoph Hertzberg, Anna C Jansen, Sergiusz Jozwiak, Rima Nabbout, Matthias Sauter, Renaud Touraine, Finbar O’Callaghan, Bernard Zonnenberg, Stefania Crippa, Silvia Comis, Guillaume Beaure d’Augères, Elena Belousova, Tom Carter, Vincent Cottin, Maria Dahlin, José Carlos Ferreira, Alfons Macaya, Mirjana Perkovic Benedik, Valentin Sander, Sotirios Youroukos, Ramon Castellana, Bulent Ulker, Martha Feucht

**Affiliations:** Sussex Kidney Unit, Royal Sussex County Hospital, Eastern Road, Brighton, BN2 5BE UK; IRCCS AUO San Martino IST–Istituto Nazionale per la Ricerca sul Cancro, Genoa, Italy; Tor Vergata University Hospital, Rome, Italy; Division of Child and Adolescent Psychiatry, University of Cape Town, Cape Town, South Africa; Associazione Sclerosi Tuberosa ONLUS, Milan, Italy; European Tuberous Sclerosis Complex Association, In den Birken, 30, 45711 Dattein, Germany; Vivantes-Klinikum Neukölln, Berlin, Germany; UZ Brussel VUB, Brussels, Belgium; The Children’s Memorial Health Institute of Warsaw, Warsaw, Poland; Hospital Necker Enfants Malades, Paris, France; Medizinische Klinik und Poliklinik IV, Klinikum der Universität München, Munich, Germany; Hôpital Nord, Saint Etienne, France; Institute of Child Health, University College London, London, UK; University Medical Center, Utrecht, Netherlands; Novartis Farma S.p.A., Origgio, Italy; Association Sclérose Tubéreuse de Bourneville, Gradignan, France; Moscow Institute of Pediatrics and Pediatric Surgery, Moscow, Russian Federation; TSA Tuberous Sclerosis Association, Nottingham, UK; Hôpital Louis Pradel, Claude Bernard University Lyon 1, Lyon, France; Karolinska University Hospital, Stockholm, Sweden; Centro Hospitalar Lisboa Ocidental, Lisbon, Portugal; Hospital Universitari Vall d’Hebron, Barcelona, Spain; SPS Pediatrična Klinika, Ljubljana, Slovenia; Tallinn Children Hospital, Tallinn, Estonia; “St. Sophia” Children’s Hospital, Athens, Greece; Novartis Pharma AG, Basel, Switzerland; Universitätsklinik für Kinder-und Jugendheilkunde, Vienna, Austria

**Keywords:** Tuberous sclerosis, Registry, Epilepsy, Subependymal giant cell astrocytoma, Angiomyolipoma

## Abstract

**Background:**

Tuberous sclerosis complex (TSC) is a rare, multisystem, genetic disorder with an estimated prevalence between 1/6800 and 1/15000. Although recent years have seen huge progress in understanding the pathophysiology and in the management of TSC, several questions remain unanswered. A disease registry could be an effective tool to gain more insights into TSC and thus help in the development of improved management strategies.

**Methods:**

**T**uber**O**us **SC**lerosis registry to increase disease **A**wareness (TOSCA) is a multicentre, international disease registry to assess manifestations, interventions, and outcomes in patients with TSC. Patients of any age diagnosed with TSC, having a documented visit for TSC within the preceding 12 months, or newly diagnosed individuals are eligible. Objectives include mapping the course of TSC manifestations and their effects on prognosis, identifying patients with rare symptoms and co-morbidities, recording interventions and their outcomes, contributing to creation of an evidence-base for disease assessment and therapy, informing further research on TSC, and evaluating the quality of life of patients with TSC. The registry includes a ‘core’ section and subsections or ‘petals’. The ‘core’ section is designed to record general information on patients’ background collected at baseline and updated annually. Subsections will be developed over time to record additional data related to specific disease manifestations and will be updated annually. The registry aimed to enrol approximately 2000 patients from about 250 sites in 31 countries. The initial enrolment period was of 24 months. A follow-up observation period of up to 5 years is planned.

**Results:**

A pre-planned administrative analysis of ‘core’ data from the first 100 patients was performed to evaluate the feasibility of the registry. Results showed a high degree of accuracy of the data collection procedure. Annual interim analyses are scheduled. Results of first interim analysis will be presented subsequent to data availability in 2014.

**Implications:**

The results of TOSCA will assist in filling the gaps in understanding the natural history of TSC and help in planning better management and surveillance strategies. This large-scale international registry to study TSC could serve as a model to encourage planning of similar registries for other rare diseases.

## Background

Tuberous sclerosis complex (TSC) is an autosomal dominant genetic disorder resulting from mutations in either *TSC1* (encoding hamartin) or *TSC2* (encoding tuberin) [1]. The disease has an estimated prevalence between 1/6800 and 1/15000 population [2-4]. Tuberous sclerosis complex can have a serious debilitating effect in affected individuals. The disease is characterised by growth of hamartomas in several organs, including the brain, kidneys, lungs, heart, eyes, and skin [1,4]. Tuberous sclerosis complex can affect people of all age groups with various organ systems involved in different ways and at varying times. The manifestations appear to have an age-related expression pattern; some manifestations are typically identifiable at birth while others develop later at different ages. Some patients may have a mild form and remain undiagnosed, whereas others may have a severe disease. Moreover, the clinical presentation of TSC may vary greatly even within a family [1,5,6].

The central nervous system is one of the most commonly and severely affected organ systems in patients with TSC. Epilepsy is seen in 70% to 90% of patients, usually begins in the first year of life, and is often refractory to treatment [7,8]. Autism spectrum disorder, intellectual disability, and other neurodevelopmental and psychiatric disorders associated with TSC also lead to a significant disability [9]. The main structural brain lesions include cortical tubers, subependymal nodules, and subependymal giant cell astrocytomas (SEGAs) [1,5]. Cortical tubers develop prenatally and are seen in 90% of patients. Subependymal nodules are seen in 80% of patients but are usually asymptomatic [10]. SEGAs are slow growing tumours seen in 10% to 15% of patients, usually manifesting in the first two decades of life [1,7]. Initially, SEGAs are asymptomatic but can grow to a considerable size and cause ventricular dilatation and hydrocephalus [8,10,11]. Renal angiomyolipomas are seen in up to 80% of patients [7]. These usually do not appear before 3 years of age [5]. Renal complications have been found to be the most frequent cause of TSC-related mortality in adult patients [12]. Pulmonary manifestation of TSC includes lymphangioleiomyomatosis (LAM) which is seen in about 40% of women of reproductive age [13]. Recently, a higher prevalence (60% to 80%) of LAM in women over 30 years of age has been reported [14]. Although symptomatic LAM is less common, it has the potential to cause severe morbidity, including pneumothorax, chylothorax, and respiratory failure and can seriously influence the prognosis and quality of life (QOL) [13-15]. Cutaneous manifestations include angiofibromas, hypomelanotic macules, shagreen patch, and ungual fibromas with each appearing at a distinct age. Hypomelanotic macules are typically detected in infancy or early childhood and shagreen patch is more common after 5 years of age. Facial angiofibromas usually appear in early childhood and progress in late childhood or adolescence. These can cause disfigurement and are, therefore, a cause of cosmetic and psychological concern [6,16]. Cardiac rhabdomyomas are present in up to 70% of infants with TSC and can be detected in prenatal ultrasound. Most do not cause clinical manifestations and regress spontaneously during the first few years of life [16,17]. Similarly, ophthalmic lesions including retinal hamartomas rarely interfere with vision [1]. In addition, lesions can occur virtually in any other organ system.

Given that symptoms are varied and none can be considered pathognomonic, making an accurate diagnosis of TSC can be a significant challenge. Specific criteria for the diagnosis of TSC have evolved over the years, as new genetic and clinical information has become available. The diagnostic criteria for TSC were first proposed in 1979 and thereafter, were revised in 1992 and 1998 [18-20]. In 2012, these guidelines were further refined at the Second International TSC Consensus Conference [21]. The panel also provided the updated surveillance and management recommendations for standardised, optimal clinical management of patients with TSC [22]. Two major changes in the most recent diagnostic criteria were the reduction of diagnostic classes from three (definite, probable, and possible) to two (definite and possible) and the inclusion of positive genetic testing as an independent diagnostic criterion. The identification of either *TSC1* or *TSC2* pathogenic mutation is now sufficient to make a definite diagnosis of TSC [21].

The discovery of the TSC genes helped in understanding the pathogenesis of TSC. Mutations in either of the two TSC genes are detected in approximately 85% of the patients. *TSC1* and *TSC2* play an important role in the normal functioning of the mammalian target of rapamycin (mTOR) pathway. The proteins encoded by these two genes form a tumour-suppressor complex, which regulates activation of the mTOR complex 1. Mutations on either of the two genes result in constitutive activation of the mTOR pathway, which in turn leads to abnormal cellular proliferation and differentiation producing the hamartomatous lesions [23]. mTOR inhibition as a treatment approach for TSC has been investigated with promising results. Everolimus, a potent, selective, orally bioavailable mTOR inhibitor, has been approved for the treatment of SEGA and renal angiomyolipoma associated with TSC [24-29]. In addition, mTOR inhibitors also appear to have a clinically beneficial effect on several other manifestations of TSC including angiofibromas, LAM, and, potentially, epilepsy [30-35].

Although numerous advances have been made in the past decade to understand TSC, many gaps still remain. The natural history of many of the manifestations, their variability and their prognostic roles are not yet completely understood. Although various manifestations appear to have an age-related expression pattern, the published data on detailed and long-term longitudinal description of disease manifestations and their changes over time are limited. Gaps also exist in understanding the following aspects: rare symptoms and co-morbidities of TSC; the relationship between genotype and phenotype; and the various interventions, treatments, and their outcomes. Further, long-term data for new intervention strategies (e.g., mTOR inhibitors) are needed. We know that TSC associated with a *TSC2* gene mutation is typically more severe than that caused by a *TSC1* gene mutation. However, the reason for this is not clear [36]. The molecular mechanism of TSC in 15% of patients with ‘no mutation identified’ is also not yet known [5].

Making an accurate diagnosis and recommending appropriate treatment and surveillance plan continue to be challenging for the treating physician. Although revised monitoring guidelines have been published recently, the TSC consensus panel acknowledged that recommendations for many interventions and management strategies were not based on a high level of evidence [22]. More information on this rare disease is required to further optimise management strategies. In 2011, Novartis in collaboration with medical experts and patient advocates evaluated the need for a TSC registry to address some of the existing gaps. Online surveys were conducted in January 2011 and February 2011, by sending questionnaires to a number of individuals and organisations in Europe that were involved in management of TSC. All the complete responses were analysed. The results from the survey and from round table discussions suggested that national TSC registries did not exist in many European countries. A need for collaboration on a larger scale, instead of solely relying on the evidence obtained from a limited number of patients was strongly felt. There was a clear consensus regarding the need to establish a registry, which resulted in the conception of **T**uber**O**us **SC**lerosis registry to increase disease **A**wareness (TOSCA). Subsequently, some non-European countries also joined the registry, thereby making TOSCA a truly global venture. The data from this registry will assist in gaining a better understanding of the clinical course of TSC, and thus, provide substantial evidence on the appropriate clinical surveillance of this disease with age-dependent manifestations. In this manuscript, we present the design and methodology of TOSCA, which, to our knowledge, is the first multi-country registry aimed at collecting information on TSC.

## Methods

### Description of TOSCA registry design

**Figure 1:**
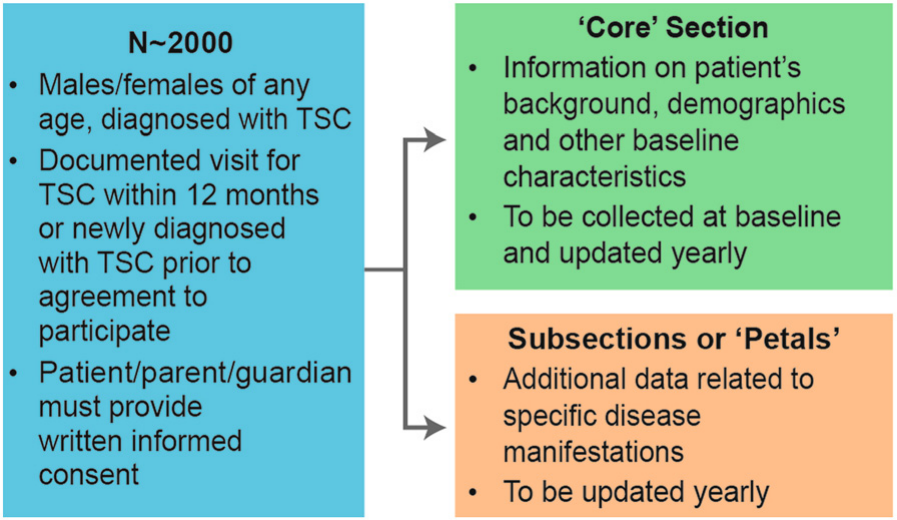
**TOSCA registry design.**

**Figure 2:**
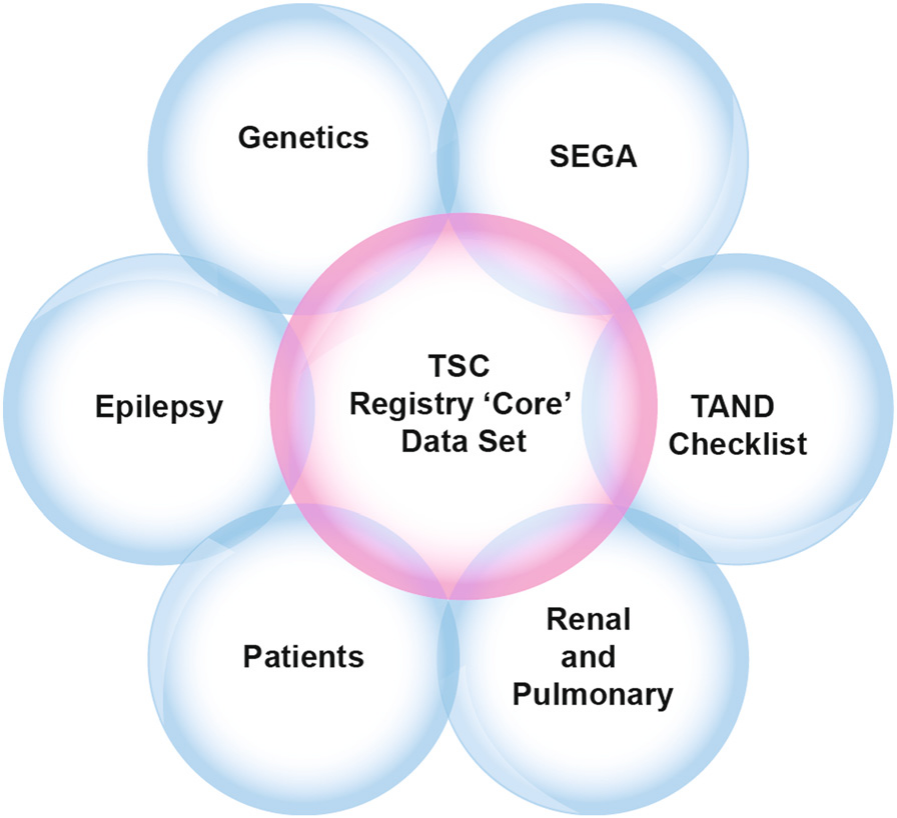
**The ‘flower and petal’ model of TOSCA registry.** Abbreviations: SEGA: Subependymal giant cell astrocytoma; TAND, TSC-associated neuropsychiatric disorders; TSC: Tuberous sclerosis complex.

TOSCA is a multicentre, international disease registry that has been designed to collect data to assess the manifestations, interventions and their outcomes in patients with TSC (Figure 1). The registry is structured retrospectively and prospectively to collect patient and disease information. TOSCA consists of the main ‘core’ section and subsections (also referred to as ‘petals’) (Figure 2). The ‘core’ section of the registry is designed to collect a general predefined set of patient background data including demographics, family history, prenatal history, and disease features (i.e., neurological, neuropsychiatric, renal, cardiovascular, pulmonary). This mandatory section will ensure that at least a minimum amount of essential information on each patient is collected to allow meaningful analyses. Additional and more detailed data related to specific disease manifestations will be collected in subsections of the registry. All participants will have ‘core’ baseline data. In addition, an annual update is required for the ‘core’ section and for subsection data, as appropriate. In order to enhance active patient partnership in this registry, a patient self-report section is designed to collect each patient’s own additional information including data on patient experience and QOL.

The data are retrieved from hospital discharge files, clinical records, clinic visits, electronic medical records, patients’ questionnaires, and *ad hoc* clinical databases. Follow-up visits are scheduled according to the standard practice of the site and as per the treating physician’s best judgement.

The data are recorded on an electronic case report form (eCRF) that is accessed via a secure web portal hosted by Quintiles (a contract research organisation). The eCRF has specific questions in English about the manifestations of TSC and results of relevant investigations and treatment. Input of data is carried out by local investigators or their deputies, and then independently checked by a network of clinical research associates for accuracy and consistency using the original local case records. The web portal has an explanatory manual to guide the investigators. Utmost care has been taken during the design of the eCRF and the explanatory manual to ensure that they are comprehensible to individuals from different participating countries where English is not the first language. Individual demographic data such as name, address, hospital, and/or health insurance number are not uploaded to the web portal so that anonymity can be maintained.

TOSCA registry is not designed as a population-based epidemiological registry; therefore, may not provide representative incidence and prevalence data on the disease and its manifestations.

The TOSCA Post-Authorization Safety Study (PASS) is a drug safety sub-study which is based on a requirement of the European Medicines Agency (EMA). The purpose of TOSCA PASS is to obtain data to assess the long-term safety and tolerability profile of everolimus (Votubia®) in approved indications for the treatment of patients with TSC residing in the European Union.

### Eligibility criteria

Patients of any age diagnosed with TSC could participate in the registry. Each patient must have a documented visit for TSC within the preceding 12 months or must be newly diagnosed with TSC prior to agreement to participate in the registry. To be eligible for TOSCA PASS, participants from TOSCA must be on everolimus (Votubia®) for an approved TSC indication in the European Union. All patients (parents or guardians, where applicable) must sign the informed consent form before any information or data is provided to the registry.

### Study duration

The first patient was entered in the registry in August 2012. The initial enrolment period was of 24 months. The patients will be followed up for up to 5 years. For paediatric patients included in TOSCA PASS, the follow-up period will be extended until they reach Tanner stage V if evaluated per local routine practice, or until the age of 16 years for females and 17 years for males, regardless of whether the therapy has ended. This prolonged follow-up period will ensure collection of long-term data on safety and on the impact of the drug on sexual maturation and potential fertility.

### Objectives and main variables

The objectives of TOSCA include mapping the course of TSC manifestations and their effects on prognosis by obtaining information such as the proportion of patients with each TSC manifestation (e.g., SEGA, renal angiomyolipoma, LAM); the presence of any relationship between manifestations affecting different organs and the time of their appearance; prevalence of single organ manifestations and their natural history. The incidence and prevalence of rare symptoms (like pancreatic neuroendocrine tumour, chordoma, perivascular epithelioid cell tumour, hemi hypertrophy, secreting pituitary adenoma, hamartoma of the stomach, scoliosis, thickening and sclerosis of the calvarium, parathyroid adenoma) and comorbidities will also be obtained. Interventions and their outcomes will be recorded with an aim of assessing their frequency by type, by the order of their use and by the role of treating physician. The outcome of each manifestation will be assessed by the type of intervention. Scientific hypotheses to be tested in preclinical and/or clinical investigations will be identified. Validated questionnaires will assess QOL of patients with TSC. The objectives of the TOSCA registry and TOSCA PASS sub-study with the main variables are listed in Tables 1 and 2, respectively.

**Table 1:** **TOSCA objectives and main variables**

**Objectives**	**Main variables**
To map the course of TSC manifestations and their prognostic roles	Proportion of patients with each TSC manifestation (e.g., SEGA, angiomyolipoma, lymphangioleiomyomatosis), its complications and overall survival
To identify patients with rare symptoms and comorbidities	Incidence and prevalence of rare symptoms and comorbidities
To record interventions and their outcomes	Frequency of interventions by type, by sequence and by role of the treating physician, and of physician specialty and referral to site of excellence
	Outcome of manifestations by type of intervention
	Frequency and type of follow-up visits, imaging/tests, hospitalisation, emergency room visits and surgical procedures
To contribute to creating an evidence base for disease assessment and therapy and inform research on TSC	Identification of scientific hypotheses to be tested in preclinical and/or clinical investigations; promote observational and experimental prospective studies on specific groups of patients
To measure quality of life in patients with TSC	Validated questionnaires on quality of life
To collect information on sexual maturation/endocrine assessments in patients with TSC, if available	Endocrine assessments (e.g., FSH, LH, Inhibin B, estradiol, testosterone, progesterone)

*Abbreviations*: *FSH* follicle stimulating hormone, *LH* luteinising hormone, *SEGA* subependymal giant cell astrocytoma, *TOSCA* **T**uber**O**us **SC**lerosis registry to increase disease **A**wareness, *TSC*, tuberous sclerosis complex.

**Table 2:** **TOSCA PASS objectives and main variables**

**Objectives**	**Main variables**
To document the long-term safety and tolerability profile of everolimus in the treatment of patients with TSC residing in the European Union who are prescribed everolimus for approved indications	Incidence of AEs, SAEs, and everolimus-related AEs in the observation period
	Incidence of events of special interest (e.g., noninfectious pneumonitis, severe infections, hypersensitivity, stomatitis, secondary amenorrhea in post-adolescent females, etc.)
To collect everolimus therapeutic drug monitoring data within routine clinical practice as per the Summary of Product Characteristics	Everolimus blood concentration, if available

*Abbreviations*: *AE* adverse event, *PASS* post-authorization safety study, *SAE* serious adverse event, *TOSCA* **T**uber**O**us **SC**lerosis registry to increase disease **A**wareness, *TSC* tuberous sclerosis complex.

### Statistical considerations

**Figure 3:**
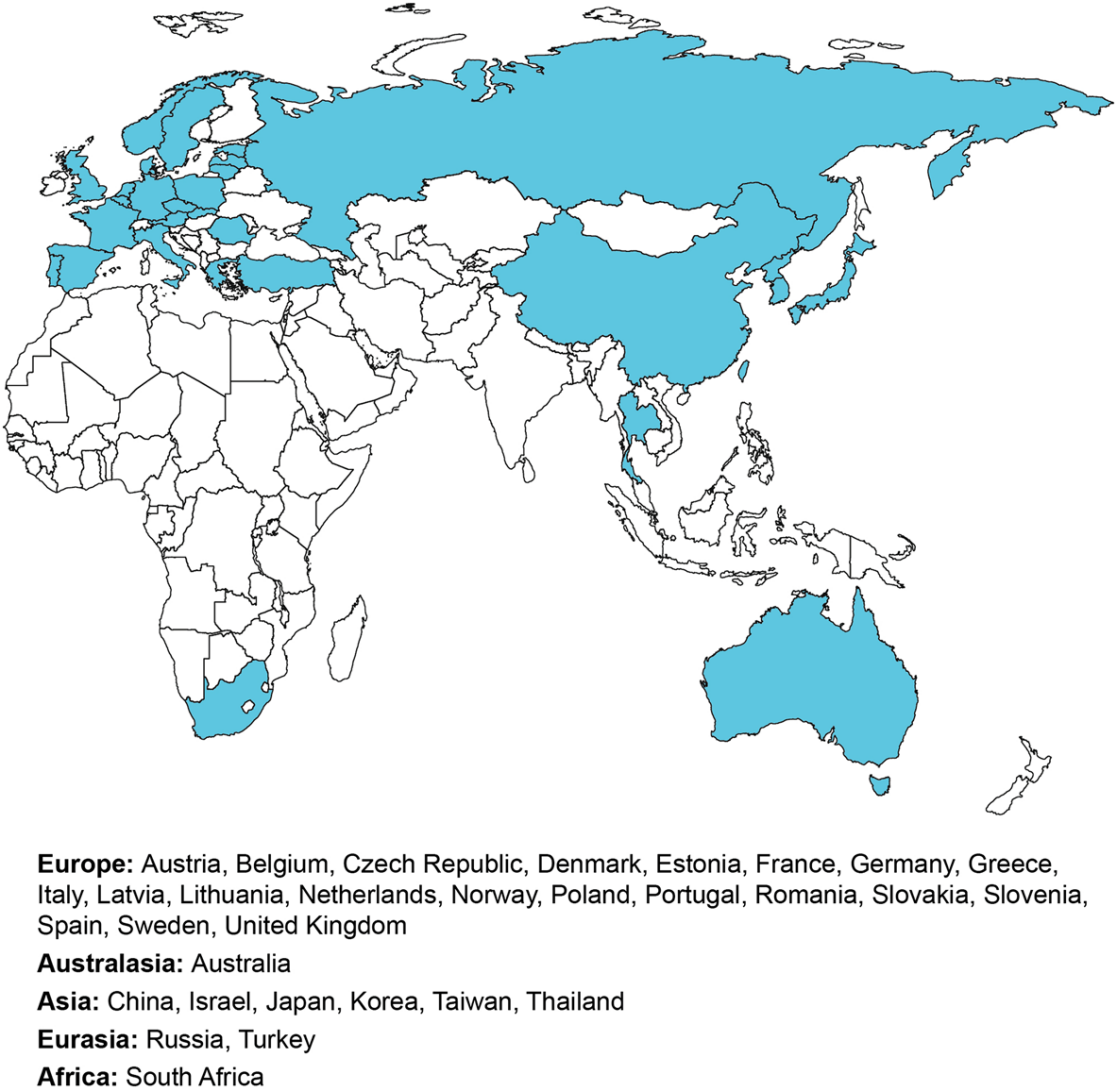
**Countries participating in TOSCA.**

It was estimated that approximately 2000 patients meeting eligibility criteria would be enrolled from about 250 sites across 31 countries in TOSCA (Figure 3). TOSCA PASS was expected to enrol at least 150 patients from about 100 sites across 15 countries. Formal sample size estimation was not required due to the descriptive nature of this registry, thus allowing flexibility in the number of patients to be enrolled.

All patients enrolled in the TOSCA disease registry will be considered in the analysis. Data will be summarised with respect to the background (demography, genotype, family history, age at diagnosis), clinical features (neurological, neuropsychiatric, renal, cardiovascular, pulmonary, dermatological), rare manifestation, and co-morbidities of each patient. Each variable will be analysed as a whole. Continuous variables will be analysed in terms of value at the time of registration and change from baseline at subsequent examinations. Categorical variables (e.g., presence/absence of a condition or manifestation) will be analysed in terms of frequency distribution at the time of registration and at follow-up. Missing data will not be imputed.

### TOSCA registry organisation

**Figure 4:**
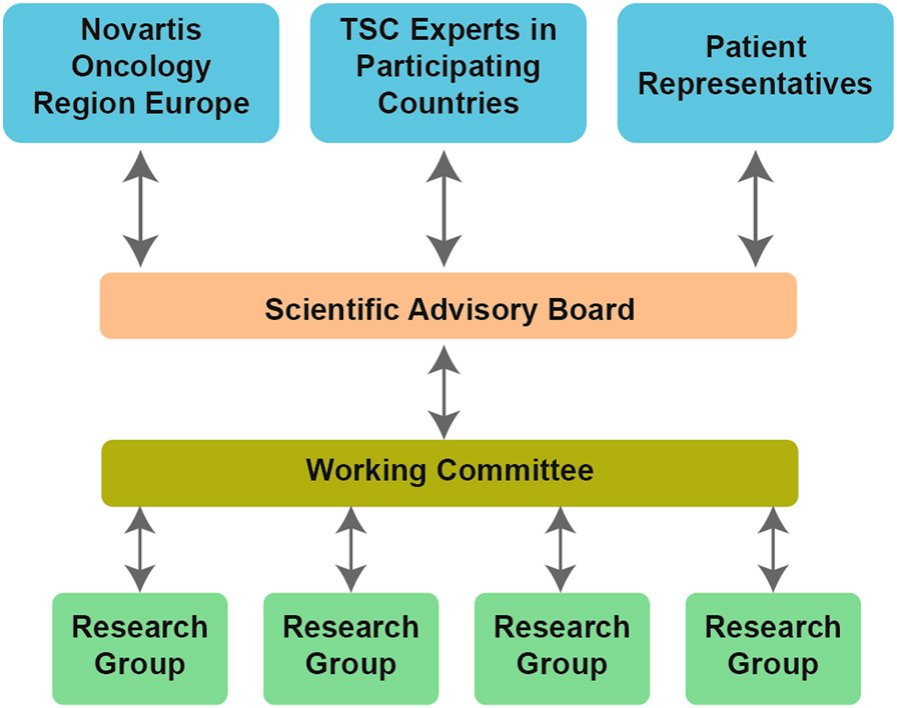
**TOSCA registry organisation.**

TOSCA registry organisation (Figure 4) includes the Scientific Advisory Board (SAB), a Working Committee, and Research Groups, which work in collaboration.

### Scientific advisory board

The Scientific Advisory Board consists of up to 30 members (TSC healthcare professionals from various specialities and patients’ association group representatives) external to the sponsor and a maximum of three representatives of the sponsor. The Scientific Advisory Board is responsible for the scientific principles of the registry, promotion of the use of the registry in the participating sites, publication of data in agreement with the publication policy, and approval of research projects. The chairperson, elected by the members of the SAB, presides over and facilitates the running of SAB meetings for a period of 12 months.

### Working committee

The Working Committee is a subgroup consisting of up to 14 members from the SAB and is responsible for the registry content and coordination of all the operational activities. The Working Committee is also responsible for defining the statistical analysis plan and publication policy, and for developing and maintaining the database structure of the registry in collaboration with other members, according to their specialty and research interests.

### Research groups

Any participating centre can submit a specific research proposal for inclusion into the TOSCA database. Letters of intent are submitted annually to the working committee, which recommends a set number of projects for potential inclusion. Accepted ‘petal’ project participants form a Research Group, which may consist of a variable number of physicians participating in the registry. Each Research Group is responsible for submission of a research project proposal to the Working Committee, and for the management and analysis of the particular project accepted into the database. Participation in Research Group projects is not mandatory for all sites.

### Publication policy

Results of the analyses will be submitted for publication and/or posted in a publicly accessible database. Publications will be based on data from all sites that are analysed. The Working Committee is responsible for development and coordination of publications and for ensuring that planned timelines are met. The Working Committee also coordinates reviews and approvals.

### Ethical compliance

Written informed consents were obtained from all patients, parents, or guardians to participate in TOSCA and TOSCA PASS. The protocol was approved by an ethics committee at each centre, before the first patient was enrolled. This study has been designed and is being implemented and reported in accordance with the Guidelines for Good Pharmacoepidemiology Practices (GPP) of the International Society for Pharmacoepidemiology (ISPE), the Strengthening the Reporting of Observational Studies in Epidemiology (STROBE) guidelines, and the ethical principles laid down in the Declaration of Helsinki.

## Results

### Administrative analysis

The first administrative analysis was performed in March 2013 on complete data collected for baseline ‘core’ data set for the first 100 patients with the aim of evaluating feasibility and accuracy of the data collection procedure. A total of 469 fields of information were evaluated for each of the 100 patients. In more than 90% of patients, information on 85% of the fields was found to be complete. The administrative analysis showed a high degree of accuracy ensuring optimum quality of the collected data. Subsequent interim analyses are planned approximately every 12 months starting from 2014. These interim analyses aim to review, analyse, and publish the findings.

### Current enrolment

Although enrolment was closed on 10 August 2014, the registry continues to recruit patients who are eligible for TOSCA PASS sub-study. This recruitment is being done to have at least 150 patients in TOSCA PASS, as per the commitment made to EMA. As of 24 September 2014, a total of 2216 patients had been enrolled in TOSCA, of which, 162 patients are also eligible for TOSCA PASS sub-study.

## Discussion

The complexity of the disease due to multisystem involvement and the difficulty in finding sufficient cases due to rarity of the disease make systematic study of TSC a real challenge. Pragmatic clinical studies have been conducted in patients with TSC but typically based on smaller sample sizes [17,24-27,32,33]. Good understanding of the natural course of the disease, which requires examining a large sample over an extended period of time, is essential to develop appropriate management strategies.

While randomised controlled trials form an important part of research and are considered the gold standard to evaluate treatment efficacy, patient registries also play an important role in describing the natural course of the disease and thus in identifying optimal disease management strategies [37]. Patient registries are helpful tools to address questions or issues that may not be addressed in clinical trials, including description of patients that cannot be included in trials. In fact, randomised controlled trials and patient registries complement each other in the evaluation of patient outcomes [37]. Patient registries are very good tools to study characteristics of a disease, its management, and outcomes with or without treatment. Registries help in improving patient care and healthcare planning especially for rare diseases and also contribute to improving social, economic, and QOL outcomes in these diseases [38].

Registries established for rare genetic diseases have helped the participants to meet others with similar conditions and also benefited the researches to gain access to huge amount of relevant information. The European Huntington’s Disease Network that was established in 2004 enrolled a large number of patients from 13 European countries, thereby demonstrating the feasibility to collect data across countries speaking different languages [39]. Also notable are TREAT-NMD (Translational Research in Europe -Assessment and Treatment of Neuromuscular Diseases) Duchenne muscular dystrophy registries, which are standardised registries for patients with Duchenne muscular dystrophy from several countries [40].

### Significance of TOSCA

This international multicentre registry has been designed to study a large cohort of patients with TSC from different ethnic groups over a prolonged period of time, to obtain answers to some specific questions, and to fill some of the existing gaps. TOSCA will investigate outcomes of interventions under natural conditions. As the data will be obtained from a large patient population, the analysis will have more power. Collaboration of an academic steering committee, a pharmaceutical company, and patient/carer organisations is a major strength of TOSCA. The registry includes a subsection/petal completely dedicated to the wider needs of the patients. Various TSC patient communities have been involved in this ‘petal’. This subsection will help to evaluate the impact of the disease on the life of families of the affected individuals. Similarly, each of the other petals will add to the existing knowledge in the respective areas.

### How does TOSCA aim to address the gaps?

The TOSCA database has been designed to provide both cross-sectional and longitudinal data on TSC. General information collected in the ‘core’ section of the registry from a large patient population worldwide will help in identifying trends. TOSCA will help to obtain data on various diagnostic and treatment strategies and study the impact of the same. We will understand whether the time and the method of diagnosis of TSC and its manifestations differ in various countries. We will know whether the methods for the various interventions including the order in which they are administered in different countries are similar. The data obtained can also help to understand whether rare manifestations are truly rare and whether certain co-morbidities are more frequent than others. Information on current use of genetic tests for diagnosis in different countries and information on affected family members (mother/father, number of relatives affected) will also be obtained. In addition, we will acquire information on patient needs and QOL.

Specific ‘subsections’ designed as research projects will collect more detailed information. Each research project has a main question that essentially is a gap in the understanding of the disease. Information from the ‘core’ section along with the additional questions will help to fill the gap. Research projects related to SEGA, renal angiomyolipoma, genetics, epilepsy, TSC-associated neuropsychiatric disorders, and patient socioeconomics questionnaires will be launched in 2014. Additional research projects (related to, skin/eye manifestations and LAM) will be analysed and may be launched at a later date.

### Significance of TOSCA PASS

Overall safety data for everolimus in TSC available from completed, controlled and uncontrolled studies indicate that everolimus is generally well tolerated. The safety profile is characterised by manageable adverse events that are generally reversible and non-cumulative [24-27]. TOSCA PASS will provide additional safety data for everolimus when used for prolonged periods in a larger number of patients, similar to phase IV studies.

### Overcoming challenges

Some of the challenges in conducting patient registries are ensuring continuous data entry, data quality, and completeness. It is important to assess the feasibility of the registry and to ensure efficiency of the data collection procedure [41]. The results of the first administrative analysis conducted for this purpose have been encouraging. The ‘core’ section was purposely designed as the mandatory section to improve completeness of the data entered in the registry. Annual data collection was selected to ensure longitudinal progression, whilst not being too cumbersome.

It is important to be mindful that TOSCA is not a population-based study. Patients are enrolled from hospital services and specialised centres and may not represent all cases of TSC. While more severe cases are likely to be over-represented, there is a probability of missing less severe cases that are undiagnosed or do not require medical care. To reduce this bias, TOSCA investigators are encouraged to seek consent for participation from all patients meeting the eligibility criteria.

Future studies must aim to cover the entire population.

## Conclusion

The results from this registry will provide better understanding of the natural history of the disease and will facilitate development of better management and surveillance strategies for patients with TSC. The information collected from this large cohort of patients with TSC will guide future research. This is a model study that could promote observational and experimental prospective studies on specific groups of patients in other rare diseases.

## Abbreviations

AE: Adverse event
EMA: European Medicines Agency
FSH: Follicle stimulating hormone
GPP: Good pharmacoepidemiology practices
ISPE: International Society for Pharmacoepidemiology
LAM: Lymphangioleiomyomatosis
LH: Luteinising hormone
mTOR: Mammalian target of rapamycin
PASS: Post-Authorization Safety Study
QOL: Quality of life
SAB: Scientific Advisory Board
SAE: Serious adverse event
SEGA: Subependymal giant cell astrocytoma
STROBE: Strengthening the reporting of observational studies in epidemiology
TAND: TSC-associated neuropsychiatric disorders
TOSCA: **T**uber**O**us **SC**lerosis registry to increase disease **A**wareness
TSC: Tuberous sclerosis complex
